# Sociodemographic characteristics and longitudinal progression of multimorbidity: A multistate modelling analysis of a large primary care records dataset in England

**DOI:** 10.1371/journal.pmed.1004310

**Published:** 2023-11-03

**Authors:** Sida Chen, Tom Marshall, Christopher Jackson, Jennifer Cooper, Francesca Crowe, Krish Nirantharakumar, Catherine L. Saunders, Paul Kirk, Sylvia Richardson, Duncan Edwards, Simon Griffin, Christopher Yau, Jessica K. Barrett

**Affiliations:** 1 MRC Biostatistics Unit, University of Cambridge, Cambridge, United Kingdom; 2 Institute of Applied Health Research, University of Birmingham, Birmingham, United Kingdom; 3 Department of Public Health and Primary Care, University of Cambridge, Cambridge, United Kingdom; 4 Nuffield Department for Women’s & Reproductive Health, University of Oxford, Oxford, United Kingdom; 5 Health Data Research, Oxford, United Kingdom; University of Edinburgh, UNITED KINGDOM

## Abstract

**Background:**

Multimorbidity, characterised by the coexistence of multiple chronic conditions in an individual, is a rising public health concern. While much of the existing research has focused on cross-sectional patterns of multimorbidity, there remains a need to better understand the longitudinal accumulation of diseases. This includes examining the associations between important sociodemographic characteristics and the rate of progression of chronic conditions.

**Methods and findings:**

We utilised electronic primary care records from 13.48 million participants in England, drawn from the Clinical Practice Research Datalink (CPRD Aurum), spanning from 2005 to 2020 with a median follow-up of 4.71 years (IQR: 1.78, 11.28). The study focused on 5 important chronic conditions: cardiovascular disease (CVD), type 2 diabetes (T2D), chronic kidney disease (CKD), heart failure (HF), and mental health (MH) conditions. Key sociodemographic characteristics considered include ethnicity, social and material deprivation, gender, and age. We employed a flexible spline-based parametric multistate model to investigate the associations between these sociodemographic characteristics and the rate of different disease transitions throughout multimorbidity development. Our findings reveal distinct association patterns across different disease transition types. Deprivation, gender, and age generally demonstrated stronger associations with disease diagnosis compared to ethnic group differences. Notably, the impact of these factors tended to attenuate with an increase in the number of preexisting conditions, especially for deprivation, gender, and age. For example, the hazard ratio (HR) (95% CI; *p*-value) for the association of deprivation with T2D diagnosis (comparing the most deprived quintile to the least deprived) is 1.76 ([1.74, 1.78]; *p* < 0.001) for those with no preexisting conditions and decreases to 0.95 ([0.75, 1.21]; *p* = 0.69) with 4 preexisting conditions. Furthermore, the impact of deprivation, gender, and age was typically more pronounced when transitioning from an MH condition. For instance, the HR (95% CI; *p*-value) for the association of deprivation with T2D diagnosis when transitioning from MH is 2.03 ([1.95, 2.12], *p* < 0.001), compared to transitions from CVD 1.50 ([1.43, 1.58], *p* < 0.001), CKD 1.37 ([1.30, 1.44], *p* < 0.001), and HF 1.55 ([1.34, 1.79], *p* < 0.001). A primary limitation of our study is that potential diagnostic inaccuracies in primary care records, such as underdiagnosis, overdiagnosis, or ascertainment bias of chronic conditions, could influence our results.

**Conclusions:**

Our results indicate that early phases of multimorbidity development could warrant increased attention. The potential importance of earlier detection and intervention of chronic conditions is underscored, particularly for MH conditions and higher-risk populations. These insights may have important implications for the management of multimorbidity.

## Abstract 2

In this multistate modelling analysis, Sida Chen and colleagues examine sociodemographic characteristics and longitudinal progression of multimorbidity using data from primary care records in England

## Author summary

**Why was this study done?:**

- Multimorbidity, the presence of 2 or more chronic conditions in an individual, is a growing concern in ageing societies. A better understanding of how these conditions develop and progress over time, and the factors associated with this process, is important for more effective management and treatment.
- Previous research has analysed the association between certain socioeconomic and behavioural factors and the rate of disease progression over time. However, these studies typically focused on a limited number of conditions and rarely considered all possible combinations. Furthermore, their analyses often rely on relatively small datasets.
- There is a gap in our detailed understanding of the impact of sociodemographic characteristics—such as ethnicity, deprivation, age, and gender—on the progression of multiple chronic conditions.

**What did the researchers do and find?:**

- We analysed the health records of over 13 million participants in England from 2005 to 2020, focusing on how factors like ethnicity, deprivation, gender, and age are associated with the accumulation of 5 common conditions: cardiovascular disease (CVD), type 2 diabetes (T2D), chronic kidney disease (CKD), heart failure (HF), and mental health (MH) conditions.
- We found that factors like deprivation, age, and gender generally have a stronger link to the diagnosis of these conditions compared to ethnicity. Moreover, the impact of deprivation, age, and gender tend to be weakened as the number of preexisting conditions a person has increases.
- In particular, when an individual already has an MH condition, and if they were older, male, or from more deprived groups, they were expected to develop other conditions like CVD, T2D, and HF more quickly compared to scenarios involving other preexisting conditions.

**What do these findings mean?:**

- Our findings suggest that early stages, when people are starting to develop multiple health issues, especially when MH problems are first diagnosed and in high-risk groups, may require more attention for improved patient care and healthcare strategies.
- Our results underscore the need to investigate and better understand the different biological, psychological, and societal factors that influence the progression to multimorbidity.
- Note that our analysis is based on health records, which may have incomplete or inaccurate information, including potential inaccuracies in condition diagnosis. These limitations may have an influence on our results.

## Introduction

Multimorbidity or multiple long-term conditions is defined as the coexistence of 2 or more chronic conditions in an individual [1]. Multimorbidity increases with age, and, because of the complexity of managing those with multiple health conditions, it is of increasing importance in countries with ageing populations [2]. Existing research indicates that multimorbidity is associated with poorer quality of life, worsened health outcomes, increased healthcare use, and higher mortality [3,4]. Multimorbidity develops sequentially, as individuals accumulate diagnoses, but to date, research has mainly described cross-sectional patterns of multimorbidity, primarily focusing on estimating multimorbidity prevalence and identifying multimorbidity clusters [5–8]. We need to understand how diseases accumulate over time and the associations of potentially important sociodemographic characteristics with this accumulation process. This may help identify those at high risk of developing further diseases. A recent systematic scoping review summarised research on longitudinal modelling and analysis of multimorbidity patterns; see [9]. One of these analyses investigated the sequence of occurrence of diseases using a visualisation approach, stratified by deprivation status, age, and ethnicity [10]. They reported that the more socially deprived had a greater risk for multimorbidity and that ethnicity was associated with the development of multimorbidity.

The progression of multimorbidity can be conceptualised as an individual transitioning through various states of comorbidity, each representing a distinct disease profile. This sequence of disease accumulation can be viewed as a specific form of event history. Multistate models (MSMs) extend the traditional survival model to encompass more than 2 states, providing a useful probabilistic framework for modelling complex longitudinal event history data and making predictive inferences. MSMs have been effectively used in recent multimorbidity studies. For instance, MSMs have been applied to investigate the progression of 3 chronic conditions in an elderly population [11] and to analyse socioeconomic inequalities in life expectancy with and without multimorbidity [12]. They have also been used to assess the influence of lifestyle factors on disease accumulation [13] and to explore the effects of sociodemographic and clinical factors on several stages of multimorbidity development [14]. Other studies have utilised MSMs to examine the potentially differing impacts of lifestyle risk factors on cardiometabolic multimorbidity [15] and to assess the role of midlife factors on different cardiometabolic disease trajectories [16]. In a more recent study, a 17-state MSM was used to examine the impact of the order of disease acquisition on patient mortality [17].

Despite the significant potential of MSMs for multimorbidity analysis, their current utilisation is often constrained by computational challenges associated with model estimation and/or constraints in sample size. Most existing MSMs for multimorbidity have been restricted to incorporate only a minimal number of conditions (for instance, 3) or represent multimorbidity status merely as counts of conditions rather than discerning specific condition combinations to simplify the model structure. In addition, current models largely rely on simple parametric models such as the Weibull hazard model, which has limited flexibility, or Cox semiparametric models, which are incapable of absolute risk quantifications or predictions over time. While certain risk factors such as age, gender, and some lifestyle factors have been considered, detailed studies on the impact of sociodemographic characteristics on the rate of disease-specific multimorbidity progression remain scarce. Furthermore, the reliance on relatively small cohort samples in most of the previous analyses may limit the power of their findings relative to the number of states of the models and subgroups.

In this work, we propose a flexible parametric multistate modelling framework for describing the temporal progression of 5 chronic conditions and investigating the association between several key sociodemographic risk factors, including age, gender, ethnicity and social and material deprivation, and the rate of different disease transitions. We focus on cardiovascular disease (CVD), type 2 diabetes (T2D), chronic kidney disease (CKD), mental health (MH) conditions, and heart failure (HF), which are cardiometabolic, renal conditions and mental conditions that are common, important, and well coded in primary care due to the Quality and Outcomes Framework (QoF) incentivisation programme for general practice. These conditions have well-established treatment and prevention strategies that are known to frequently co-occur, especially in socioeconomically deprived areas [18]. By treating different disease combinations as separate states, our model comprises a total of 33 states and 112 transitions and is estimated based on a large dataset of approximately 13 million English electronic primary care records. Importantly, our MSM extends previous works by allowing for more comorbidity states and disease transition types, thus providing a more detailed characterisation of the impacts of the sociodemographic factors on the progression of these 5 diseases. While the framework is flexible and capable of accommodating different or additional conditions in principle, our primary analysis centres on these 5 conditions to avoid overcomplicating the presentation and interpretation of results.

## Methods

### Ethics statement

The Clinical Practice Research Datalink (CPRD) Research Database has ethics approval from the Health Research Authority to support research using anonymised patient data (East Midlands—Derby Research Ethics Committee, reference 21/EM/0265). Data were approved for this study by the CPRD Independent Scientific Advisory Committee (protocol 19_265).

### Data sources

The study investigated the temporal sequence of diagnosed chronic conditions in a large anonymised dataset of electronic primary care records, the CPRD [19]. The CPRD Aurum database contains routinely collected primary care electronic health records from 19 million participants across the 738 English general practices that use EMIS Web software [19] (10% of practices and 13% of the English population). It is representative of the English population in terms of age, gender, regional geographical spread, and deprivation. It contains information on individual participant demographics. Clinical observations, diagnoses, test results, immunisations, lifestyle factors, and prescriptions are recorded as Read Version 2, SNOMED Clinical Terms, and EMIS Web clinical codes. Data are available from 1995 onwards, and there is a median follow-up of 4.2 years (IQR: 1.5 to 11.4) for all patients and 9.1 years (IQR: 3.3 to 20.1) for currently registered patients [19].

### Conditions

In this study, we placed our attention on 5 specific chronic conditions: (1) CVD, which comprises ischaemic heart disease (IHD) and stroke or transient ischaemic attack (TIA); (2) T2D; (3) CKD; (4) MH conditions, comprising depression and/or anxiety; and (5) HF. Within CVD, we distinguished between IHD, which primarily affects the heart, and stroke or TIA, which primarily affects the brain. For MH conditions, we concentrated on depression and anxiety as they are the most prevalent MH conditions [20], and, while there is clinical overlap in their presentation, they are typically phenotypically quite distinct from other less common MH conditions such as psychotic disorders, posttraumatic stress disorder, and substance misuse disorders. The presence of a chronic condition was determined by the occurrence of one of the relevant clinical codes (code lists are provided in S1 Code Lists) in the participant records (based on primary care data only), and the date of diagnosis was taken as the date of the first occurrence of any associated clinical codes. An absence of the code was considered to indicate an absence of the respective condition.

### Sociodemographic characteristics

Sociodemographic characteristics include ethnicity, social and material deprivation, age (calculated from the year of birth), and sex (male/female). Ethnicity was initially classified into 5 different groups, including White, South Asian (South Asian or British Asian), Black (Black African, Black Caribbean, or Black British), Mixed, and Others (ethnicity codings are provided in S1 Code Lists) [21]. In our multistate analysis described below, Mixed was merged into Others due to their infrequency in many disease transitions. Social and material deprivation were approximated using the English Index of Multiple Deprivation (IMD), which is derived from a range of factors including income, employment, education, crime, and living environment. The IMD is a widely accepted, area-based composite measure of area-level deprivation [22]. In the raw CPRD Aurum data, each participant was assigned an IMD decile, ranging from 1 (least deprived) to 10 (most deprived), based on their area of residence. For our analysis, we consolidated IMD deciles into quintiles to facilitate the presentation of our results. This level of granularity seemed sufficient for our purpose, as we are primarily interested in the general association patterns between deprivation levels and disease progression.

### Cohort description

Participants were eligible for inclusion in the study if they were registered in a general practice contributing to CPRD between the study start date and the study end date. The study start date was defined as the latest of the date the participant turned 18, 01/01/2005, or 6 months after the participant registration start date. The study end date was defined as the earliest of the participant registration end date, date of death, or the last collection date for practice (11/05/2020). The minimum period of registration ensures time for participant characteristics to be recorded and prior diagnoses to be transferred/recorded. Participants were followed up continuously during the study period. For conditions where code lists were combined the earliest valid code in any code list was used as the date of incidence, and long-term conditions recorded before the study start date were treated as prevalent. In preparing our dataset for analysis, we began by excluding participants aged over 90 years at the study start date (*n* = 194,726). From the remaining participants, we further excluded those with gender classified as indeterminate (*n* = 395), those with missing data on IMD (*n* = 40,564), and those with missing ethnicity information (*n* = 6,345,105). Additionally, participants with no follow-up period (*n* = 1,199,267) were removed from the dataset. The final cohort consists of a total of 13.48 million participants. More details regarding the sample selection procedure are provided in Fig A in S1 Text.

### Statistical method

**Fig 1:**
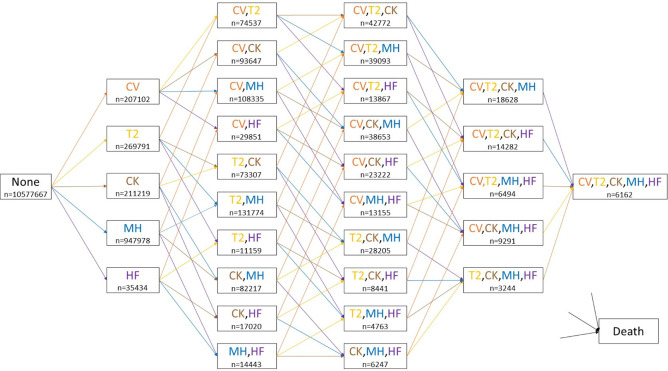
The multistate diagram depicting disease progression. The initial state, labelled “None,” represents participants with no comorbidities at the start of the study. Intermediate comorbidity states are colour coded as follows: cardiovascular disease (CV, orange), type 2 diabetes (T2, yellow), chronic kidney disease (CK, brown), mental health conditions (MH, blue), and heart failure (HF, purple). Each coloured arrow indicates a transition, with the colour corresponding to the disease that is diagnosed when the transition takes place (for instance, orange arrows indicate transitions into a CVD diagnosis). The terminal state, “Death,” is directly reachable from all other states. The “n” below each comorbidity state denotes the total number of participants who entered that state during the study period.

To evaluate how sociodemographic characteristics such as ethnicity, deprivation, age, and sex are associated with the rate of different disease accumulation during multimorbidity development, we considered an MSM with a total of 33 states and 112 allowable direct transitions (see Fig 1 for an illustration). The states incorporate all possible combinations of the 5 conditions a person may have during the process of disease progression and the permitted instantaneous state-to-state transitions were indicated by a direct solid line with an arrow indicating the direction of the transition. The death state is the terminal state and can be directly reached from any other state. There are 2 simplifying assumptions underlying the multistate diagram shown in Fig 1. The first assumption is that once a chronic condition is diagnosed, it remains “switched on.” This practice is common in large-scale health research and is also necessitated by the limitations of our available data. The second assumption is that no more than 1 event per person (either a diagnosis or death) can happen on the same day. This is based on the observation that “simultaneous transitions”—two or more events happening on the same day—are extremely rare. Among our cohort of 13.47 million participants, the most frequent instances of such transitions did not exceed about 5,000 cases (i.e., less than 0.04%). In fact, these same-day diagnoses are likely to be artificially increased by same-day diagnostic tests or recording of diagnoses, which raises questions about the accuracy of the timing of these recorded transitions. Therefore, we excluded the data associated with these simultaneous transitions from our analysis.

To flexibly specify the MSM, we followed the suggestions of [23] to use a separate time-to-event model for each transition, and we used a clock-reset time scale (i.e., the time scale resets to zero after every transition) so that the outcome of interest is the time from one disease diagnosis to the next diagnosis or death. More specifically, we modelled the logarithm of the cumulative transition-specific intensities as a spline function of log time [24]. This allowed us to estimate hazard ratios (HRs) for each factor—age, sex, ethnicity, and deprivation—adjusted for the other factors (for instance, HRs for the ethnic group are adjusted for age, sex, and IMD). To limit the model’s complexity and facilitate summarisation and consistent interpretation of results, we assumed proportional hazards and no interactions between these predictors for each transition. When fitting each transition, we considered data from all participants who underwent that transition during the study period. Any diagnoses that occurred before the study’s start date were treated as prevalent. Model parameters were estimated and their uncertainties quantified based on maximum likelihood estimation (MLE) theory, and the *p*-values for the HRs were determined based on the Wald test. We were able to do this efficiently by leveraging the factorization property of the model likelihood, which enabled us to fit each transition independently and thus exploit parallel computing. See Text A in S1 Text for additional details. The goodness of fit of the fitted model was assessed based on the Cox-Snell residuals; see Text B in S1 Text for further details. In our implementation, we utilised the flexsurv package [25] within the R software environment. These computations were performed on the Cambridge Service for Data Driven Discovery (CSD3) High-Performance Computing (HPC) system using the Ice Lake CPUs.

To investigate the potential association between the number of preexisting conditions and the HRs associated with each sociodemographic characteristic for transitions into each of the 5 conditions, we further conducted a meta-regression analysis based on the fitted MSM (see Text C in S1 Text). The dependent variable in our meta-regression is the estimated log-transformed HR(s) for a given characteristic, pertaining to a specific transition, and the independent variable is the number of preexisting conditions prior to the corresponding transition. To account for heterogeneity among various types of transitions, we incorporated a random effect into the model. The analysis was implemented using the metafor R package [26].

To assess the robustness of our results under various modified settings, we conducted a series of sensitivity analyses. First, we evaluated the impact of condition settings on the results. We substituted HF, which is more similar to CVD among the 5 conditions, with another condition that affects a different organ system—here, we choose chronic obstructive pulmonary disease (COPD). Subsequently, we expanded the multistate transition network by incorporating COPD as the sixth condition, in addition to the 5 selected conditions. We also evaluated the impact of condition definitions, which are relevant to CVD and MH conditions as they are a composite of multiple subconditions. We considered the following 4 settings with modified definitions of CVD or MH. For CVD, we considered narrower definitions based on (1) conditions specifically affecting the brain, i.e., comprising only stroke or TIA codes; and (2) conditions specifically affecting the heart, i.e., including only the IHD code. Similarly, for MH, we considered narrower definitions based on (1) anxiety only or (2) depression only. To assess the impact of changing the resolution of deprivation groups, we considered fitting each transition using the original IMD deciles. Additionally, we examined the impact of data exclusion on the inferences (here, we focused on missing ethnicity as it accounts for the vast majority of missing data). We expanded our cohort to include participants with missing ethnicity information, assigning them to an artificial ethnic group labelled “missing.” In each scenario considered above, we refitted the model following the same procedure described above and examined the estimated relationships between sociodemographic variables and disease transitions. Lastly, to ensure our results are robust across different modelling approaches, we considered an alternative popular approach by specifying the MSM using transition-specific semiparametric Cox models. HRs for each transition were estimated in a parallel fashion as above using the “survival” package [27] in R.

## Results

### Summary statistics

The cohort baseline characteristics are shown in Table 1. At study entry, 78.5% (*n* = 10,577,667) of the eligible participants had no diagnosis of any of the 5 conditions of interest. By the end of the follow-up, 83.7% (*n* = 8,856,098) of these had not developed any of the 5 conditions, 13.8% (*n* = 1,462,966) had developed one, 2% (*n* = 209,237) had developed two, 0.4% (*n* = 42,504) had developed three, 0.06% (*n* = 6,399) had developed four, and 0.0044% (*n* = 463) had developed five of the conditions. During the study period, 5% of the study population (*n* = 670,753) died. Detailed summary statistics for each comorbidity state in our MSM are presented in Table A in S1 Text. A summary of the incidence rate for each disease transition in our MSM is provided in Table B in S1 Text. The incidence rates of transitions to the first morbidity were notably lower compared to the other transitions, and, generally, the incidence rates for transitions increased with the number of comorbidities. It should be noted that the relatively lower incidence rates for transitions from MH compared to other disease transitions could be due to the fact that patients in the MH state were generally younger (this age adjustment is incorporated into our MSM analysis).

**Table 1:** Baseline sociodemographic characteristics of study participants.

Cohort population		13,476,121
Sex (n (%))	Male	6,315,448 (46.9%)
	Female	7,160,673 (53.1%)
Age at cohort entry (median [IQR])		35 [25, 51]
Ethnicity (n (%))	White	10,753,663 (79.8%)
	South Asian	1,460,750 (10.8%)
	Black	741,504 (5.5%)
	Mixed	257,651 (1.9%)
	Others	262,553 (1.9%)
Index of multiple deprivation (n (%))	1—least deprived	2,384,659 (17.7%)
	2	2,530,868 (18.8%)
	3	2,620,501 (19.4%)
	4	3,139,349 (23.3%)
	5—most deprived	2,800,744 (20.8%)
Follow-up period (median (IQR))		4.71 [1.78, 11.28]

### Association between ethnicity and the rate of disease progression

**Fig 2:**
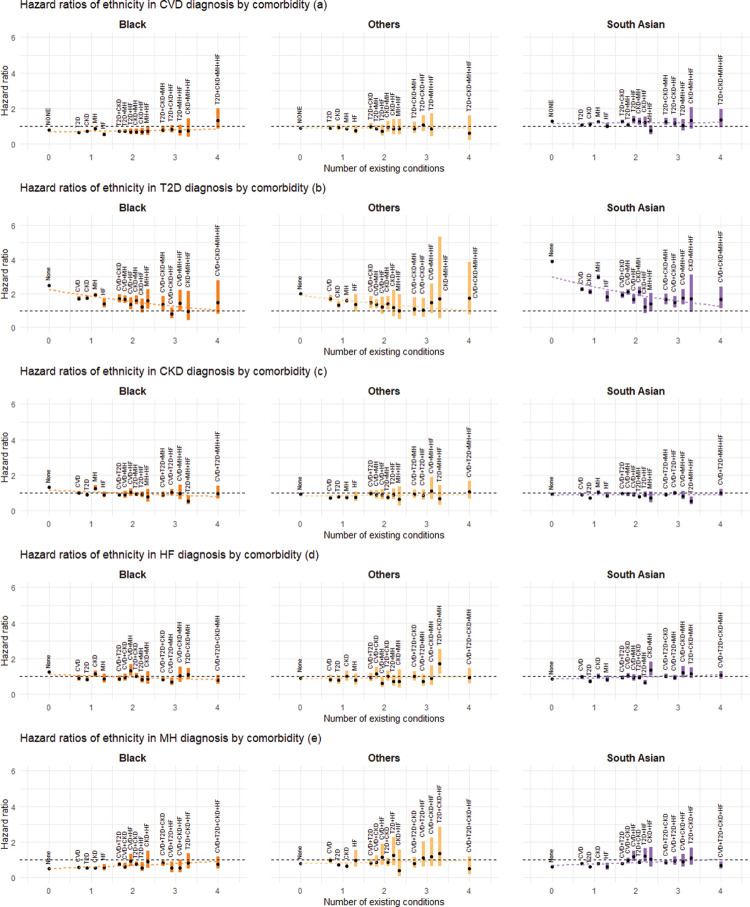
Estimated association between ethnicity and the rate of next disease transition. Panels (a) to (e) show the HRs for the association of non-White ethnic groups with the diagnosis of CVD, T2D, CKD, HF, and MH, respectively, grouped by the number of existing conditions and comorbidity status. White ethnicity is treated as the reference category. Estimated HRs are shown by black dots, with the associated 95% CIs represented by coloured bands. For transitions into each condition, the superimposed dotted lines represent meta-regression fits based on the corresponding HRs for each ethnicity category. Abbreviations: CI, confidence interval; CKD, chronic kidney disease; CVD, cardiovascular disease; HF, heart failure; HR, hazard ratio; MH, mental health conditions; T2D, type 2 diabetes.

There was no consistent association pattern between ethnicity and disease accumulation, and this relationship was modified by comorbidity status (Fig 2 and Table C.1 in S1 Text). For CVD diagnosis across different comorbidity groups (Fig 2A), HRs tended to be slightly higher for South Asian but lower for Black compared to White. For T2D (Fig 2B), HRs for South Asian, Black, and Other ethnicities were significantly greater than 1 for most comorbidity groups when compared to White. This was especially pronounced for the South Asian group, with HRs (95% CI; *p*-value) ranging from 1.20 ([0.83, 1.74]; *p* = 0.34) to 3.91 ([3.87, 3.95]; *p* < 0.001). Furthermore, these HRs generally decreased with an increasing number of preexisting conditions, as indicated by the fitted regression line from the meta-regression. In contrast, for CKD (Fig 2C), HF (Fig 2D), and MH (Fig 2E) conditions, there were minor differences between the other ethnic groups and White across different transitions, with the HRs mostly close to 1. For MH, HRs for Black compared to White were lower than 1 across different comorbidity groups, with HRs ranging from 0.52 ([0.52, 0.53]; *p* < 0.001) to 0.92 ([0.64, 1.33]; *p* = 0.66). As observed for T2D, the differences between Black and White ethnicities tended to decrease as the number of comorbidities increased.

### Association between deprivation and the rate of disease progression

**Fig 3:**
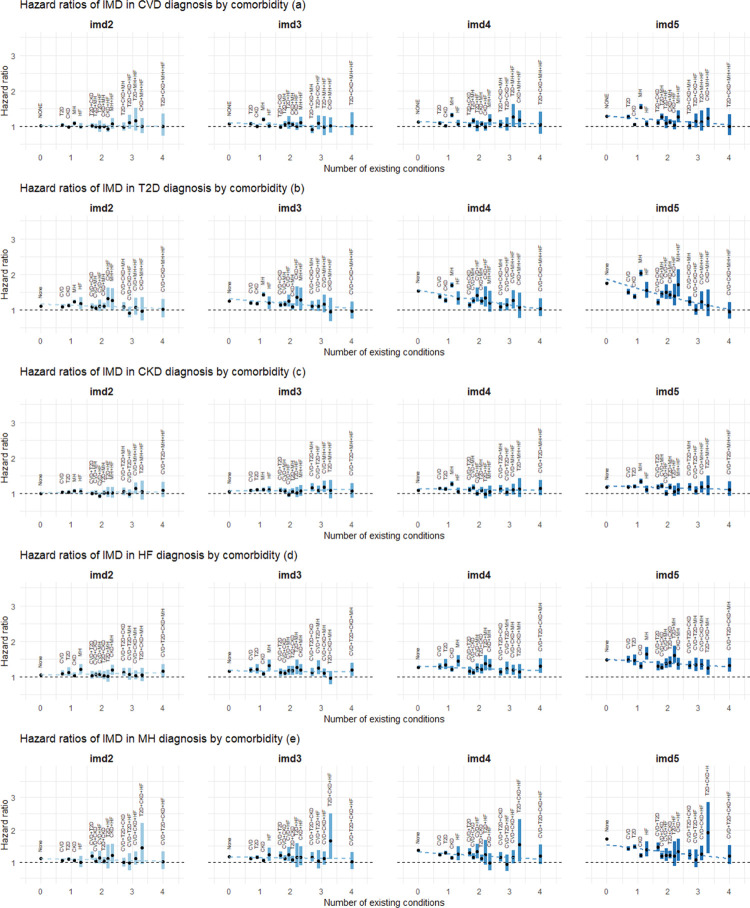
Estimated association between deprivation and the rate of next disease transition. Panels (a) to (e) show the HRs for the association of second to fifth IMD quintiles with the diagnosis of CVD, T2D, CKD, HF, and MH, respectively, grouped by the number of existing conditions and comorbidity status. The first IMD quintile (the least deprived group) is treated as the reference category. Estimated HRs are shown by black dots, with the associated 95% CIs represented by coloured bands. For transitions into each condition, the superimposed dotted lines represent meta-regression fits based on the corresponding HRs for each IMD category. Abbreviations: CI, confidence interval; CKD, chronic kidney disease; CVD, cardiovascular disease; HF, heart failure; HR, hazard ratio; IMD, the English Index of Multiple Deprivation; imd2–imd5, second–fifth IMD quintiles; MH, mental health conditions; T2D, type 2 diabetes.

Across all types of disease progression, HRs for the more deprived quintiles (second to fifth IMD quintiles) compared to the least deprived quintile (first IMD quintile) were mostly greater than 1 (Fig 3 and Table C.2 in S1 Text). As the level of deprivation increased, the HRs typically increased, indicating a higher rate of disease transition. The magnitude of this association was generally larger for transitions into T2D (Fig 3B) and HF (Fig 3D). For T2D, HRs (95% CI; *p*-value) for the fifth IMD quintile ranged from 0.95 ([0.75, 1.21]; *p* = 0.69) to 2.03 ([1.95, 2.12]; *p* < 0.001). For HF, the HRs for the fifth IMD quintile varied between 1.25 ([1.05, 1.48]; *p* = 0.01) to 1.65 ([1.48, 1.84]; *p* < 0.001). In contrast, the association was weaker for CKD (Fig 3C), MH (Fig 3E), and CVD (Fig 3A). The fitted meta-regression line further suggested that for CVD, T2D, and MH, the association between greater deprivation and rate of disease accumulation tended to be attenuated with an increasing number of comorbidities. However, this pattern was less evident for CKD and HF diagnosis.

### Association between gender and the rate of disease progression

**Fig 4:**
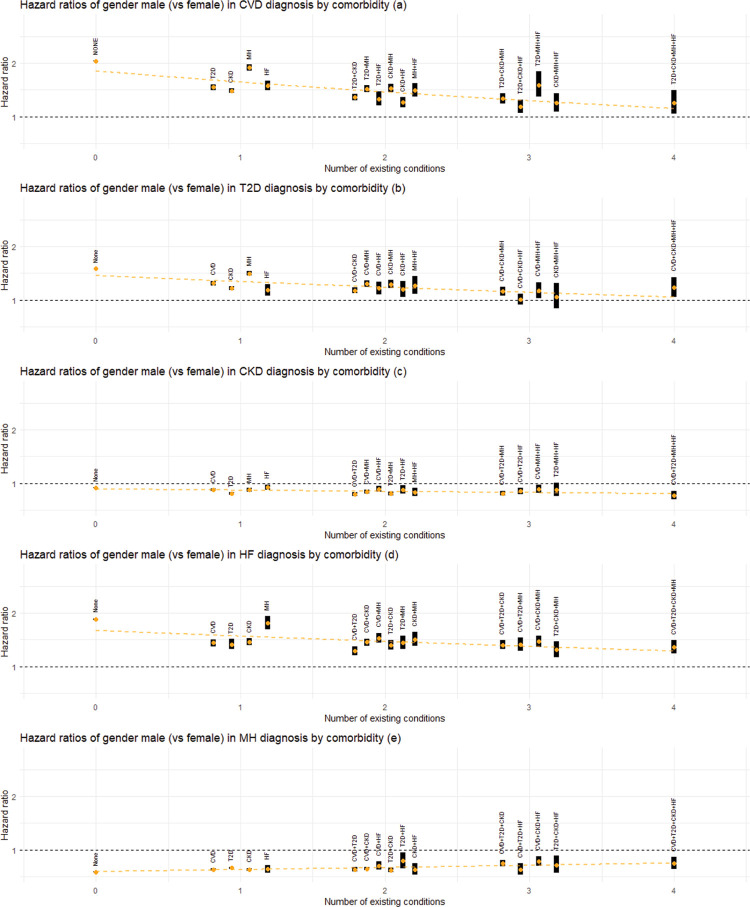
Estimated association between gender and the rate of next disease transition. Panels (a) to (e) show the HRs for the association of gender (male) with the diagnosis of CVD, T2D, CKD, HF, and MH, respectively, grouped by the number of existing conditions and comorbidity status. The female gender is treated as the reference category. Estimated HRs are shown by orange dots, with the associated 95% CIs represented by black bands. For transitions into each condition, the superimposed dotted line represents meta-regression fit based on the corresponding HRs. Abbreviations: CI, confidence interval; CKD, chronic kidney disease; CVD, cardiovascular disease; HF, heart failure; HR, hazard ratio; MH, mental health conditions; T2D, type 2 diabetes.

HRs for men, compared to women, were higher than 1 for diagnoses of CVD (Fig 4A), T2D (Fig 4B), and HF (Fig 4D) across different comorbidity groups (see also Table C.3 in S1 Text). Specifically, for CVD, HRs (95% CI; *p*-value) ranged between 1.19 ([1.07, 1.32]; *p* = 0.002) to 2.04 ([2.04, 2.05]; *p* < 0.001); for T2D, HRs were between 1.01 ([0.91, 1.11]; *p* = 0.88) and 1.59 ([1.59, 1.59]; *p* < 0.001); and for HF, they ranged from 1.29 ([1.21, 1.38]; *p* < 0.001) to 1.89 ([1.87, 1.90]; *p* < 0.001). In contrast, the HRs for CKD (Fig 4C) and MH (Fig 4E) diagnoses were lower than 1, with CKD HRs ranging from 0.77 ([0.69, 0.86]; *p* < 0.001) to 0.92 ([0.87, 0.97]; *p* = 0.004) and MH HRs from 0.58 ([0.58, 0.59]; *p* < 0.001) to 0.79 ([0.65, 0.95]; *p* = 0.01). The meta-regression analysis revealed that for CVD, T2D, and HF diagnoses, the HRs for men exhibited a downward tendency towards the null as the number of comorbidities increased, while for MH diagnosis, the initially lower HRs for men showed a slight upward adjustment with an increasing number of comorbidities. However, for CKD diagnosis, there was little difference between men and women regardless of the number of preexisting conditions.

### Association between age and the rate of disease progression

**Fig 5:**
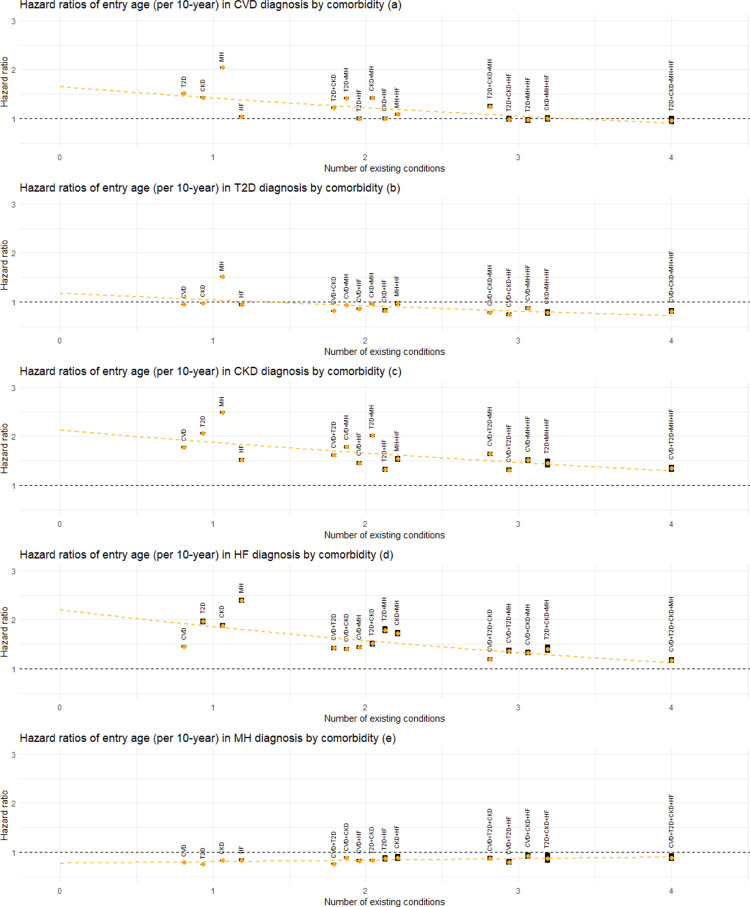
Estimated association between age and the rate of next disease transition. Panels (a) to (e) show the HRs for the association of age at entry to the current state (per 10 years) with the diagnosis of CVD, T2D, CKD, HF, and MH, respectively, grouped by the number of existing conditions and comorbidity status. Estimated HRs are shown by orange dots, with the associated 95% CIs represented by black bands. For transitions into each condition, the superimposed dotted line represents meta-regression fit based on the corresponding HRs. Abbreviations: CI, confidence interval; CKD, chronic kidney disease; CVD, cardiovascular disease; HF, heart failure; HR, hazard ratio; MH, mental health conditions; T2D, type 2 diabetes.

A 10-year increase in age was associated with an elevated rate of CVD (Fig 5A), CKD (Fig 5C), or HF (Fig 5D) diagnosis (see also Table C.4 in S1 Text). For CVD, HRs (95% CI; *p*-value) ranged from 0.97 ([0.89, 1.06]; *p* = 0.45) to 2.04 ([2.02, 2.06]; *p* < 0.001); for CKD, HRs were between 1.31 ([1.27, 1.36]; *p* < 0.001) and 2.48 ([2.46, 2.51]; *p* < 0.001); and for HF, they ranged from 1.17 ([1.11, 1.24]; *p* < 0.001) to 2.39 ([2.34, 2.45]; *p* < 0.001). This increase was especially pronounced for patients with a single preexisting condition, particularly MH, and tended to approach the null as the number of comorbidities increased. Conversely, HRs for a 10-year age increase were lower than 1 for MH (Fig 5E) diagnosis (HRs ranging from 0.75 ([0.74, 0.76]; *p* < 0.001) to 0.92 ([0.86, 0.98]; *p* = 0.02)), and for T2D (Fig 5B) diagnosis except for the transition from MH (HRs ranging from 0.74 ([0.71, 0.78]; *p* < 0.001) to 0.98 ([0.97, 0.99]; *p* = 0.001)). As comorbidities accumulated, the initially lower HRs for age showed a slight upward tendency towards 1 for MH diagnosis, while an opposite pattern was observed for transitions into T2D, where the HRs seemed to diverge further from 1 as the number of conditions increased.

### Mental health

Figs 2–5 (see also Table C in S1 Text) consistently show that the association between deprivation, gender, and age and the progression to multimorbidity tends to be more pronounced (in the direction of increasing the rate of transition) when transitions emerge from an MH condition compared to other comorbidity states. For age and deprivation (particularly age), this pattern is observed for all transitions from MH. For instance, HRs (95% CI; *p*-value) for age were 2.04 ([2.02, 2.06]; *p* < 0.001), 2.48 ([2.46, 2.51]; *p* < 0.001) and 2.39 ([2.34, 2.45]; *p* < 0.001) for transitions into CVD, CKD, and HF, respectively. For transitions into T2D, the association with age becomes larger than 1 when the comorbidity status is MH, with an HR of 1.52 ([1.50, 1.53]; *p* < 0.001), whereas it is less than 1 otherwise. The adverse association of being male (versus female) with transitions to CVD, T2D, or HF is amplified in cases involving a MH comorbidity, with HRs being 1.92 ([1.86, 1.97]; *p* < 0.001), 1.50 ([1.46, 1.54]; *p* < 0.001), and 1.82 ([1.70, 1.95]; *p* < 0.001), respectively. For ethnicity, this pattern is only seen for transitions to T2D, where the HR for South Asian ethnicity (compared to White) for the transition from MH is 2.97 ([2.86, 3.09]; *p* < 0.001).

### Sensitivity analysis results

Fig C in S1 Text shows the HRs for each sociodemographic characteristic, focusing on transitions into CVD or MH based on the respective narrower definitions specified in the statistical methods section. Modifying the conditions leads to some discrepancies in HRs, particularly for CVD. For instance, the gender (male) association for CVD diagnosis was stronger when focusing solely on IHD, while the age association was more pronounced for stroke/TIA. Despite this, the key patterns from our main results persisted, such as the attenuation of the HRs for deprivation, gender (male), and age with an increasing number of preexisting conditions.

Results of the multistate analysis with COPD replacing HF are shown in Fig D in S1 Text. Focusing on the disease transitions involving only CVD, T2D, CKD, and MH conditions, we observed no noticeable differences from the results shown in Figs 2–5. Fig E in S1 Text shows the results of the multistate analysis with COPD included as the sixth condition, focusing on the same transition types as in Figs 2–5. We see that the HRs are robust to the addition of a new condition in the transition network.

Fig F in S1 Text shows the HRs of IMD deciles, and a comparison with Fig 3 (HRs of IMD quintiles) reveals very similar association patterns in more deprived groups. The larger CIs with the former is a result of the smaller sample size per decile category. The associations between ethnicity, age and gender, and disease progression remained essentially unaltered by using IMD deciles instead of quintiles. In fact, there is statistical evidence supporting the use of IMD quintiles rather than deciles, as the Akaike information criterion (AIC) favoured the former for the majority of the transitions.

Fig G in S1 Text shows the HR results for the MSM fitted with an expanded cohort that includes those with missing ethnicity information (due to the computational time limitation of the HPC, we were not able to obtain HRs for transitions from “None”). The key patterns remained unchanged, and there were no substantial differences in the HRs when compared with Figs 2–5 in the main paper. Fig H in S1 Text shows the HR results for the MSM applied to our study cohort, specified based on semiparametric Cox models. These results are substantively the same as those obtained under our spline-based parametric model (Figs 2–5).

## Discussion

### Main findings

In this study, we examined the associations between ethnicity, deprivation, gender, and age, and the rate of progression of 5 major chronic conditions, namely, CVD, T2D, CKD, MH, and HF, using a cohort of more than 13 million English adults. Our results revealed a complex interplay between these key sociodemographic characteristics and the progression of chronic diseases, with the strength and pattern of associations varying according to disease transition types. Deprivation, gender, and age generally demonstrated stronger associations with the diagnosis of a condition than ethnic group differences. For some conditions, the impact of ethnicity, deprivation, gender, and age on the rate of transition attenuated with an increasing number of preexisting conditions. For example, the rate of developing T2D is much higher in the most socially deprived groups and in those of South Asian and Black ethnicity compared to the reference groups, but the difference becomes smaller for those with 4 chronic conditions. This pattern also applies to the diagnosis of CVD or T2D in men, and the diagnosis of CVD, CKD, or HF at younger ages. In addition, the impact of deprivation, gender, and age on the rate of diagnosis of CVD, T2D, CKD, or HF was typically more pronounced when transitioning from a single mental health condition compared to a single physical health condition or other composite conditions. The robustness of our findings was confirmed through various sensitivity analyses.

### Contribution to the literature

Our findings align with and reinforce established epidemiological evidence. For instance, our observation of a higher rate of progression to T2D in patients of South Asian and Black ethnicities, compared with the White UK population [28]. Similarly, our findings regarding a higher rate of CVD progression in South Asian ethnicity and a lower rate in the Black population echo the patterns reported in the same study [28]. Our results show that higher levels of deprivation are generally associated with an elevated rate of disease progression across all conditions and transitions. This is consistent with previous research findings, which showed that multimorbidity occurred 10 to 15 years earlier and physical–mental comorbidity was over twice as prevalent in the most socioeconomically deprived areas of Scotland than in the least deprived areas [18]. With regard to gender, our results align with previous studies, indicating men generally have an increased risk of progression to CVD, T2D, and HF but exhibit a lower risk of MH problems and CKD [20]. Moreover, our study further corroborates well-established findings that ageing is strongly associated with an increased rate of CVD, CKD, and HF [29].

Our multistate analysis offers novel insights by revealing a detailed association between sociodemographic characteristics and disease progression (CVD, T2D, CKD, MH, and HF), thereby underlining a complex dependency on the underlying comorbidity status prior to the transition. In particular, we found that the association between these characteristics and disease transitions tended to be greater when the number of preexisting conditions was lower. This implies that the impact of these characteristics may be larger during initial multimorbidity development and decrease in later stages. This pattern was particularly noticeable for ethnicity (for transitions into T2D), deprivation (for transitions into CVD, T2D, and MH), age (for transitions into CVD, CKD, MH, and HF), and gender (for transitions into CVD, T2D, MH, and HF). With regard to ethnicity and deprivation, T2D stood out from the other 4 conditions due to a stronger influence/attenuation of these factors by the number of preexisting conditions on T2D diagnosis. A plausible explanation for this pattern could be that as patients accumulate more conditions, the cumulative effect of these existing conditions may become the predominant factor influencing further disease accumulation. Additionally, enhanced health management and monitoring, which may include lifestyle modifications and medical interventions/treatments, could counterbalance the impact of sociodemographic factors.

Another novel finding is that the adverse impact of sociodemographic factors such as deprivation, gender, and age on the rate of transitioning to the next condition (CVD, T2D, CKD, or HF) tended to be more pronounced when transitioning from MH alone compared to other single or composite conditions. This pattern may again be partly attributed to biological and behavioural factors. Patients with MH as their starting condition tend to fall into the younger age groups (see Table A in S1 Text) and mental ill health is associated with unhealthy lifestyle habits such as smoking, alcohol, or substance abuse among others [30], which are known to be important risk factors for cardiometabolic diseases and contribute to the worsening of multimorbidity [13,31]. On the other hand, this adverse influence of MH appears to diminish with the presence of additional comorbidities, which could be due to similar reasons mentioned earlier.

### Strengths and limitations

This study benefits from a large dataset from primary healthcare that is broadly representative of the English population. The 5 chronic conditions considered in this study are included in an incentive scheme for the management of chronic conditions, which means they are likely to be more accurately coded [32]. We employed a flexible multistate model to comprehensively examine the association between key sociodemographic characteristics and the rate of different disease transitions during multimorbidity accumulation involving the 5 specified conditions. The model achieved a good overall fit for the vast majority of the transitions, demonstrating the utility and computational feasibility of the approach. To our knowledge, this is the largest cohort and the highest number of chronic conditions and disease transitions analysed using a flexible multistate modelling framework.

While our primary analysis centres on 5 specific conditions, our modelling and estimation approach can be adapted to accommodate different or additional conditions. The complexity of the corresponding MSM (under the same assumptions on the permitted direct transitions as described in the Methods section) grows exponentially with N, the total number of conditions under consideration. However, this rise in complexity does not imply a proportional increase in computational costs. By leveraging a valid parallel computing strategy, we estimated each transition independently and observed that for the same dataset, including more conditions typically leads to fewer observed events per transition (due to the increase in competing events for each transition). In our sensitivity analysis, we were able to fit an MSM that included a sixth condition, COPD, within our computational budget, and, importantly, the results of interest remained robust with the inclusion of COPD. We anticipate similar outcomes with the inclusion of other conditions. Nevertheless, depending on the scope of transitions of interest, a more complex MSM could pose challenges in summarising, presenting, and interpreting results. On the other hand, our sensitivity analysis indicates that the estimated MSM could be influenced to some extent by modifications to the condition definition. This is expected, given that we define the diagnosis time of a composite condition (such as CVD and MH) as the first occurrence of any associated subconditions. Therefore, any change in the set of subconditions could have a direct and complex impact on a patient’s event history data. In our sensitivity analysis, we observed greater differences in results when changing the definition of CVD compared to MH. This is partly due to the smaller overlap between CVD subtypes (stroke/TIA and IHD) as opposed to MH subtypes (anxiety and depression). In addition, we observed a closer alignment of results from the main analysis when using CVD (IHD only) or MH (depression only) definitions. This is because in our dataset, the presence of IHD and depression contributed to a larger proportion of the diagnosis of CVD and MH (using our original definitions), respectively, compared to the alternative definitions. While there are no universally accepted definitions for complex chronic conditions like CVD or MH, we believe that their determination should be considered in the context of the research question of interest and the data available.

It should be pointed out, however, that potential diagnostic inaccuracies in primary care records, such as underdiagnosis, overdiagnosis, or ascertainment bias of chronic conditions, could influence our results. For instance, diseases like CKD and T2D are often asymptomatic in the early stages, and diagnosis may occur during routine health check/screening or following the diagnosis of a related condition [33,34]. It is noted that the uptake of such checkups is generally less than 50%, with gender and ethnic disparities in the presentation to healthcare—specifically, lower uptake among men and some ethnic groups [35,36]. For instance, for CKD, renal function testing is less common in minority ethnic groups [37], and for MH, certain groups (for instance, those in the Black or South Asian ethnic groups) are less likely to present to the GP even when symptoms have developed [38]. The influence of these potential inaccuracies might be more pronounced during the early phases of multimorbidity, as participants with existing health conditions could be more likely to be invited for (and perhaps more inclined to undergo) further monitoring and screening for other health conditions. We note that for MH in particular, studies have reported significant challenges with both under- and overreporting of depression and anxiety in primary care records. Other studies have used algorithms involving prescription data, symptom, and diagnosis clinical codes to improve case detection of MH conditions [18,39,40], whereas we only used diagnosis codes. A preprint study using the same coding strategy in this same database has compared the prevalence of depression in the CPRD Aurum database with the prevalence reported in a representative sample of the English population in the Adult Psychiatric Morbidity Survey (APMS) [20,41]. Prevalence of depression in >16 year olds in CPRD Aurum was similar (19.5%; 95% CI: 19.5 to 19.5) to the doctor diagnosed depression prevalence in APMS (20.9%; 95% CI: 20.0 to 21.8) but lower than the prevalence of symptoms of depression (27.8%; 95% CI: 26.9 to 28.8). On the other hand, we acknowledge that participants who are less likely to present to primary care or receive diagnostic labels, such as socially deprived younger males, may have absent MH data. This could lead to an underrepresentation of participants from deprived communities. If anything, this may suggest that our findings—such as the association between deprivation and the rate of transition to the next condition is typically more pronounced when transitioning from a mental health condition—may actually be understated.

There are some other limitations with this study. For instance, the ethnicity categories used in this study crudely group together communities with diverse cultural practices and sociodemographic characteristics, which may mask differences in outcomes within each category. Note also that time-varying factors associated with the disease progression, such as treatment information, lifestyle modifications, and change in deprivation status, are not available from the present dataset, and, thus, they were not taken into account in the present analysis. Finally, despite our large cohort, which provides a reasonable amount of data for estimating each transition, we observe a substantial decrease in the number of transitions as more conditions accumulate. This pattern is reflected in the widening confidence interval bands in Figs 2–5 and is particularly noticeable with categorical variables like ethnicity, where an imbalance between groups exists. Consequently, this adds some level of uncertainty to our interpretations. For multimorbidity studies using a multistate model like ours, longer follow-up periods and/or larger datasets would always be desirable.

### Implications for policy and practice

The higher rates of development of the first chronic conditions in a number of identifiably disadvantaged population groups has 2 implications. First, it suggests that the factors contributing towards differences in the development of chronic conditions precede engagement with health services. This points towards wider determinants of health, particularly in relation to T2D and CVD. A better understanding of the differential impacts of socioeconomic factors on the development of chronic conditions would be valuable. Second, it implies that the greatest potential to reduce inequalities in multimorbidity between more and less socially deprived groups and between those of White and South Asian or Black ethnicities is early in the disease trajectory. Prevention of CVD and T2D might be an appropriate focus. Initiatives such as the Marmot cities are consistent with both these implications [42]. Evaluation of interventions to prevent the development of the first and second chronic conditions would be valuable. As multimorbidity, in particular mixed physical and mental health conditions, increases the use of secondary care services, an understanding of the economic effects of multimorbidity could help identify prevention priorities [42–44].

### Future directions

Clinical research implications of this analysis are to investigate and better understand the biopsychosocial factors driving the progression to multimorbidity. The greatest differences in transition rates to multimorbidity between different groups are in the development of the first few conditions, which suggests that the early stages of multimorbidity development could be particularly important and require increased attention.

Future work could apply this method to analysing the trajectories of different clusters of diseases. These might include other common conditions such as cancer, asthma, and hypertension or rarer, less explored conditions. It could also investigate groups of potentially related conditions such as MH problems, autoimmune conditions, or medically unexplained symptoms. This would help broaden the scope of multimorbidity research. From a methodological perspective, the MSM framework we utilised can be extended to address additional questions pertinent to longitudinal multimorbidity research. Ongoing work is developing a scalable and flexible joint modelling framework for both the longitudinal and multistate processes [45]. This will facilitate the investigation of associations between concurrently measured longitudinal covariates (such as biomarkers) and the progression of multimorbidity. Another direction involves the integration of modern deep learning techniques into MSM to enhance its flexibility and predictive accuracy for microsimulation. This approach will enable the generation and analysis of longitudinal multimorbidity trajectories at both individual and population levels.

## Conclusions

Multimorbidity is a growing public health challenge, and there is a need to shift from a single disease-oriented approach to a more individualised and holistic strategy. In this study, we examined the association between key sociodemographic characteristics and the progression of multimorbidity involving 5 common chronic conditions, namely, CVD, T2D, CKD, HF, and MH. Our findings reveal distinctive association patterns across different disease transitions. Notably, the impact of sociodemographic factors tends to attenuate with an increasing number of preexisting conditions, with patterns varying depending on the specific diagnosed condition. Mental health, as a comorbidity, showed unique patterns, with the impact of deprivation, gender, and age typically more pronounced when transitioning from an MH condition. These findings underscore the potential importance of earlier detection and intervention of chronic diseases, especially MH conditions, and particularly for those in the higher-risk populations. These insights may have important implications for improved patient care, socioeconomic considerations, and healthcare strategies.

## Supporting information

S1 Text Supporting information for the main manuscript. Fig A. Flowchart for sample selection. Text A. The multistate modelling approach. Text B. Model checking. Text C. Meta-regression. Table A. Summary of disease states by sociodemographic characteristics. Table B. Summary of disease transitions. Table C. Estimated hazard ratios, 95% confidence intervals, and *p*-values from the main multistate analysis. Fig B. Plots of the estimated cumulative hazard of the Cox–Snell residuals. Fig C. Estimated association between each sociodemographic characteristic and the rate of CVD or MH diagnosis based on their respective narrower definitions. Fig D. Results of the multistate analysis obtained with COPD replacing HF. Fig E. Results of the multistate analysis with COPD included as the sixth condition. Fig F. Estimated association between deprivation (IMD deciles) and the rate of disease transition by number of existing conditions and comorbidity status. Fig G. Results of the multistate analysis obtained based on the expanded cohort including patients with missing ethnicity information. Fig H. Results of the multistate analysis with transitions specified via semiparametric Cox models. COPD, chronic obstructive pulmonary disease; CVD, cardiovascular disease; HF, heart failure; IMD, the English Index of Multiple Deprivation; MH, mental health conditions.
(DOCX)

S1 Code Lists Code lists for ethnicity and the conditions considered in our study. (ZIP)

## Abbreviations

AIC: Akaike information criterion
APMS: Adult Psychiatric Morbidity Survey
CKD: chronic kidney disease
COPD: chronic obstructive pulmonary disease
CPRD: Clinical Practice Research Datalink
CSD3: Cambridge Service for Data Driven Discovery
CVD: cardiovascular disease
HF: heart failure
HPC: High-Performance Computing
HR: hazard ratio
IHD: ischaemic heart disease
IMD: Index of Multiple Deprivation
MH: mental health
MLE: maximum likelihood estimation
MSM: multistate model
QoF: Quality and Outcomes Framework
TIA: transient ischaemic attack
T2D: type 2 diabetes
